# Identification of ICU Patients with High Nutritional Risk after Abdominal Surgery Using Modified NUTRIC Score and the Association of Energy Adequacy with 90-Day Mortality

**DOI:** 10.3390/nu14050946

**Published:** 2022-02-23

**Authors:** Kyoung Moo Im, Eun Young Kim

**Affiliations:** 1 Department of Surgery, Seoul St. Mary’s Hospital, Banpo-daero 222, Seocho-gu, Seoul 06591, Korea; nicholas_im@naver.com; 2 Department of Surgery, Division of Trauma and Surgical Critical Care, Seoul St. Mary’s Hospital, College of Medicine, The Catholic University of Korea, Banpo-daero 222, Seocho-gu, Seoul 06591, Korea

**Keywords:** mNUTRIC score, mortality, surgery, intensive care unit, energy adequacy

## Abstract

For patients undergoing abdominal surgery, malnutrition further increases the susceptibility to infection, surgical complications, and mortality. However, there is no standard tool for identifying high-risk groups of malnutrition or exact criteria for the optimal target of nutrition supply. We aimed to identify the nutritional risk in critically ill patients using modified Nutrition Risk in the Critically Ill (mNUTRIC) scores and assessing the relationship with clinical outcomes. Furthermore, we identified the ideal target of energy intake during the acute postoperative period. A prospective observational study was conducted. mNUTRIC scores and the average calories prescribed and given were calculated. To identify the high-risk group of malnutrition, receiver operating characteristic curves were plotted. The ideal target of energy adequacy and predisposing factors of 90-day mortality were assessed using multiple logistic regression analyses. A total of 206 patients were analyzed. The cutoff value for mNUTRIC score predicting 90-day mortality was 5 (Area under the curve = 0.7, 95% confidence interval (Cl) 0.606–0.795, *p <* 0.001). A total of 75 patients (36.4%) were classified in the high mNUTRIC group (mNUTRIC ≥ 5) and had a significantly higher postoperative complication and longer length of surgical intensive care unit stay. High mNUTRIC scores (odds ratio = 2.548, 95% CI 1.177–5.514, *p =* 0.018) and energy adequacy less than 50% (odds ratio = 6.427, 95% CI 1.674–24.674, *p =* 0.007) were associated with 90-day mortality.

## 1. Introduction

Patients who undergo abdominal surgery usually have alterations in the structural barrier of the gastrointestinal tract or the absorptive ability of nutrients. In addition, surgeons’ concern about the firmness of surgical anastomosis can limit the early initiation of enteral feeding in these patients. Consequently, after abdominal surgery, patients are easily predisposed to malnutrition [1]. Hence, identifying critically ill patients who are at risk of malnutrition after abdominal surgery and providing adequate nutritional support would be important [2]. Many guidelines, including the American Society for Parenteral and Enteral Nutrition (ASPEN), the Society for Critical Care Medicine, and the European Society for Clinical Nutrition and Metabolism (ESPEN), suggest early nutritional intervention for patients admitted to a surgical intensive care unit (SICU) [3,4]. However, many studies reported that significant gaps between guidelines and practice were commonly observed among surgical patients in intensive care units (ICUs) [5,6]. Moreover, it is difficult to collectively specify the target patients for nutritional support among those who have undergone various types of surgery on different organs.

In 2011, Heyland et al. [7] developed the Nutrition Risk in the Critically Ill (NUTRIC) score, specifically aimed at identifying critically ill patients who would most likely benefit from nutritional supplementation. The NUTRIC score consists of age, Acute Physiology and Chronic Health Evaluation II, Sequential Organ Failure Assessment score, number of comorbidities, days from hospital admission to ICU admission, and serum interleukin-6 (IL-6). However, IL-6 is not easy to measure on a daily basis and is not commonly used in most hospitals. Therefore in 2015, Rahman et al. [8] developed and validated the NUTRIC score without serum IL-6 (modified NUTRIC score). It became a more practical and easier-to-use tool based on the variables that are easily obtained in the critical care setting. Although several studies have validated its diagnostic value as a predictor of malnutrition [9], the results of these studies were mostly limited to patients receiving only medical treatment [10,11,12,13,14]. In contrast, relatively little information is available for patients who underwent abdominal surgery, despite their having a high risk of malnutrition, which affects the postoperative prognosis.

Herein, we analyzed the nutritional status based on mNUTRIC scores and identified the predictors of 30-, 60-, and 90-day mortality in patients after abdominal surgery. Moreover, we demonstrated the association between mortality and different nutritional support targets in patients with a high risk of malnutrition after abdominal surgery.

## 2. Participants and Methods

### 2.1. Patient Enrollment and Data Collection

From March 2019 to February 2020, a prospective observational cohort study was conducted in the SICU of a tertiary referral hospital. The data were obtained from the electronic medical record, operative reports, and nursing charts. Patients admitted to the SICU for more than 48 h after abdominal surgery were eligible for study enrollment. Patients were enrolled regardless of the method of surgery, either open, laparoscopy, or robotic surgery. The patients were excluded if they were (1) aged under 18 years, (2) underwent surgery under local anesthesia, (3) pregnant, (4) discharged or expired within 48 h of ICU admission, (5) readmitted to the ICU, (6) diagnosed with liver failure, (7) diagnosed with renal failure and on renal replacement therapy, or (8) lacked individual data to calculate the mNUTRIC score.

The collected data included demographics and the laboratory profiles of nutritional status such as total protein, albumin, pre-albumin, transferrin levels, and cholesterol profile. Sequential organ failure assessment (SOFA) scores and mNUTRIC scores were only calculated at the time of SICU admission. Daily nutritional delivery data were recorded for all participants, including the feeding strategy and type and amount of nutrients received by the patients. The total daily calorie and protein prescribed or delivered for each patient was calculated by checking enteral feeding pump history, flowsheet documentation, and calorie count. Calorie and protein intake were monitored during the entire ICU stay and stopped at ICU discharge. We defined energy adequacy (%) as the total calories delivered divided by the total calories prescribed, multiplied by 100. The 30-, 60-, and 90-day mortality values were defined as any mortality developed within 30-, 60-, and 90-days after surgery as either an inpatient or outpatient, respectively. Any complication graded III, IV, or V on the Clavien–Dindo classification [15] was considered a postoperative complication.

The current study was approved and carefully monitored by the Institutional Review Board of the Ethics Committee of our institution (IRB No. KC21RISI0869). Informed consent was obtained from all individual participants or guardians of the participants.

### 2.2. Nutritional Assessment by Modified NUTRIC Scores and Nutritional Supplement Strategies

As previously described by Rahman et al. [8], the mNUTRIC score was calculated at the time of SICU admission from five variables that included age, the Acute Physiology and Chronic Health Evaluation II score, the SOFA score, the number of comorbidities, and the days from hospital to ICU admission. The total mNUTRIC scores ranged from 0 to 9 points. Receiver operating characteristic curve analysis was used to express the ability of mNUTRIC scores to predict 90-day mortality by the area under the curve (AUC). The appropriate cutoff was identified as the highest combined sensitivity and specificity using Youden’s index. With an appropriate mNUTRIC cutoff score for predicting 90-day mortality, all participants were divided into high and low mNUTRIC groups for further analysis. The same method was also used to select an optimal threshold energy adequacy value in the high mNUTRIC group.

For the participants, in principle of standard care, the total calorie requirements were calculated as 25–30 kcal/kg/day and 1.2–1.5 g/kg/day protein as in the ASPEN and ESPEN guidelines [3,16]. Usual body weight was used for the calculations, but the ideal body weight was used for obese patients who had a body mass index > 25 kg/m^2^. The use of propofol and glucosaline was calculated as energy intake. All patients received a volume-based feeding protocol with stomach feeding unless the patients had a high risk of aspiration. Gastric residual volumes were measured every 6 h, and if the residual volume was higher than 500 mL/6 h, the attending physician may have delayed the enteral feeding. However, in this situation, we also examined the abdomen for intolerance, and when there was no sign of acute abdominal complications, we usually applied prokinetics or other medication rather than stopping the feeding.

Under our institution’s policy, if the attending physician suspected that a patient was at high risk of malnutrition or if the fasting period was expected to be more than seven days following abdominal surgery, a consultation with the nutritional support team (NST) was conducted. The NST is a multidisciplinary support team comprised of physicians, nurses, dietitians, and pharmacists who assess the nutritional status of patients, determine their nutritional needs, and give recommendations for nutritional therapy [17,18]. The high and low mNUTRIC groups were additionally subcategorized into two groups according to the presence of NST implementation.

### 2.3. Statistical Analysis

All statistical analyses in this study were conducted using SPSS statistical package software (version 24.0 for Windows; SPSS, Inc., Chicago, IL, USA). The categorical variables were analyzed using the chi-squared test or Fisher’s exact test. Continuous data are expressed as the median value with range or the mean ± standard deviation (SD), and the overall differences were assessed by the Student’s t-test. Variables were tested for normal distribution using the Kolmogorov–Smirnov test, and in the case of variables not normally distributed, the Mann–Whitney test was used. The primary outcome was the nutritional status in the high and low mNUTRIC groups and the correlation between nutritional and clinical outcomes after surgery. The secondary outcome was to assess the ideal target of nutrition supplementation using energy adequacy in surgical patients with high mNUTRIC scores. Only significant variables in univariate analysis were used for multiple regression analysis using the Cox proportional hazard model, presented by the relative risk with a corresponding 95% confidence interval (CI). The differences were regarded as statistically significant for *p*-values of <0.05.

## 3. Results

**Figure 1:**
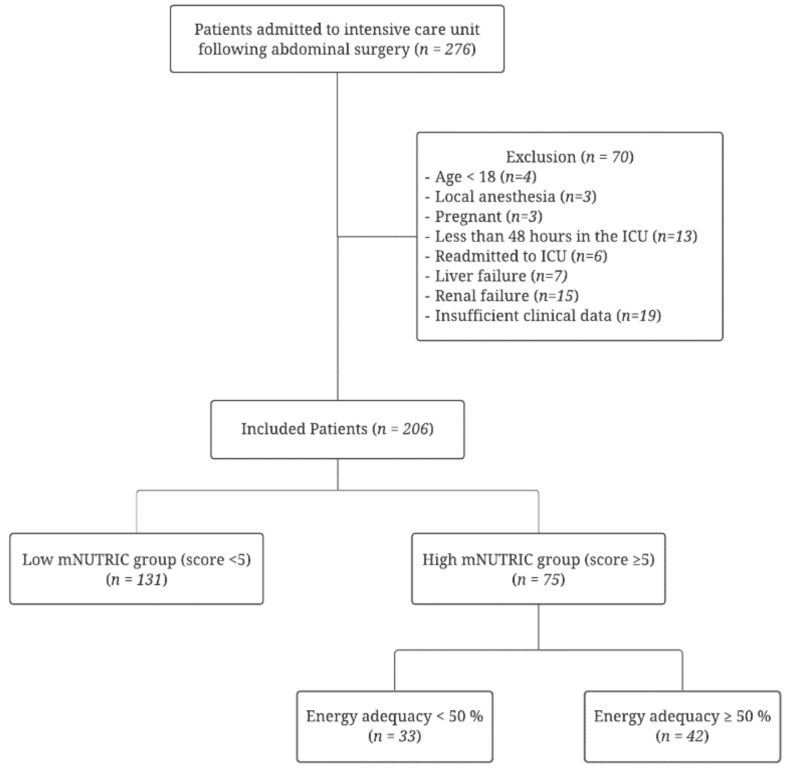
The schematic diagram of study enrollment.

During the study period, a total of 276 patients were admitted to the SICU following abdominal surgery. According to the exclusion criteria, 70 patients were excluded, and 206 patients were finally analyzed. We classified the patients into two groups: those with mNUTRIC scores of ≥5 as the high mNUTRIC group (75 patients, 36.4%) and those with scores of <5 as the low mNUTRIC group (131 patients, 63.6%) (Figure 1).

**Figure 2:**
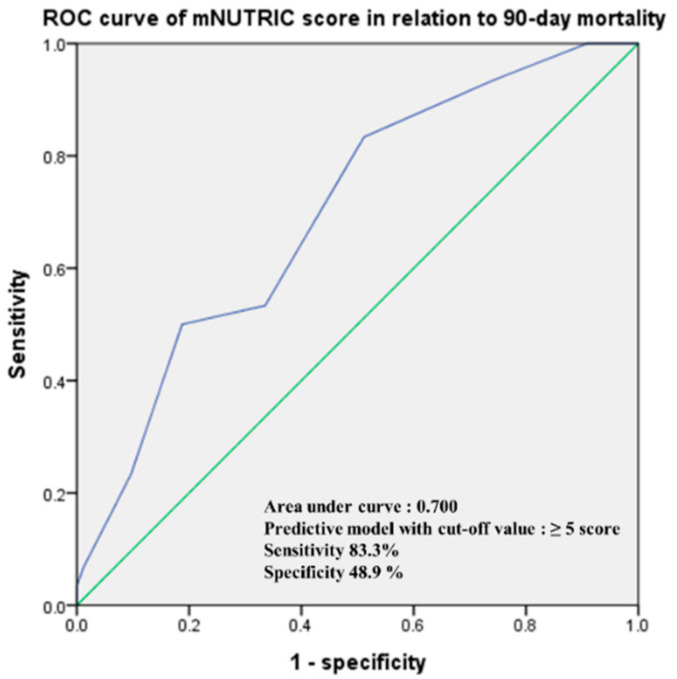
Receiver operating characteristic (ROC) curve of the logistic regression model of mNUTRIC scores in relation to 90-day mortality in total participants.

As depicted in Figure 2, mNUTRIC scores revealed a sufficient prognostic potential in predicting 90-day mortality in critically ill patients after abdominal surgery (AUC = 0.700, 95% CI: 0.604–0.795, *p <* 0.001). The highest combined sensitivity and specificity was found with a cutoff of 5 (sensitivity = 83.3%, specificity = 48.9%). The baseline characteristics of the enrolled patients and comparative analysis between the low mNUTRIC and high mNUTRIC groups are presented in Table 1. In the total participants, the mean age was 62.5 (range, 26–91), and 143 patients (69.4%) were male. The mean mNUTRIC score was 4 (range, 0–9), and 30 (14.6%) patients died within 90 days after surgery. Patients in the high mNUTRIC group showed significantly higher postoperative complication rates (36 versus 20.6%, *p =* 0.021) than the low mNUTRIC group. There were also significant differences between the two groups in the length of ICU stay, the length of hospital stay, and laboratory parameters related to nutrition. Ninety-day mortality was more frequently observed in the high mNUTRIC group (16 cases, 21.3%) than in the low mNUTRIC group (14 cases, 10.7%, *p =* 0.042). Regarding the type of nutrition that the patients received, 40 patients (19.4%) received parenteral nutrition only, 9 patients (4.4%) received enteral nutrition only, and 20 patients (9.7%) received parenteral nutrition and enteral nutrition concomitantly. The remaining 137 patients (66.5%) received an oral diet, and for each type of nutrition, there was no significant difference between the two groups.

**Figure 3:**
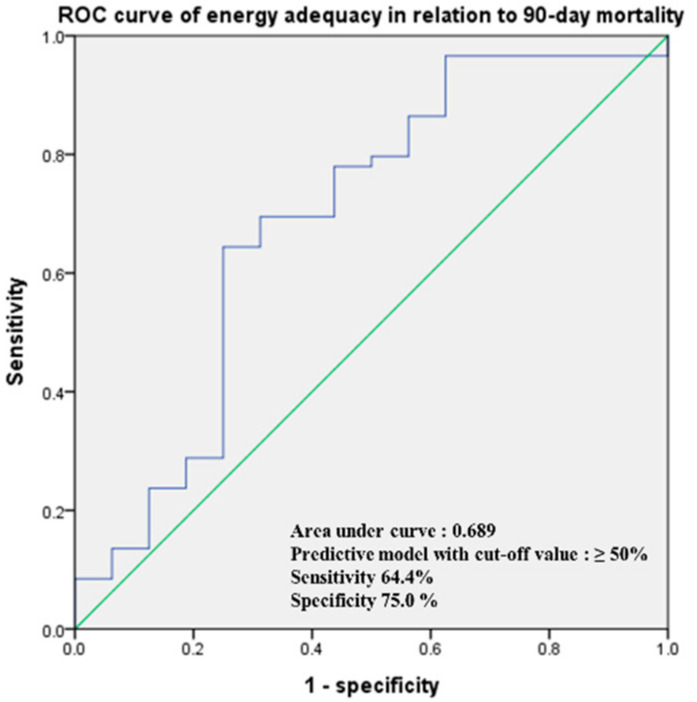
Receiver operating characteristic curve of the logistic regression model of energy adequacy in the high mNUTRIC group in relation to 90-day mortality.

As shown in Figure 3, we performed a receiver operating characteristic curve analysis of energy adequacy in the high mNUTRIC group for predicting the 90-day mortality risk. The 90-day mortality prediction by energy adequacy showed an AUC of 0.689 (95% CI: 0.525–0.852, *p =* 0.021), and the best cutoff value was at 50% (sensitivity = 64.4%, specificity = 75.0%). Therefore, the nutritional status and clinical outcomes were compared according to the energy adequacy of 50% and also assessed according to energy adequacies of 60% and 70%, as described in previous reports [19,20]. In the high mNUTRIC group, 33 patients (44%) had energy adequacies of less than 50%. Using energy adequacy cutoffs of either 50%, 60%, or 70%, there was no difference in the length of stay, postoperative complications, or 90-day mortality between the groups. However, the 90-day mortality was significantly higher in the high mNUTRIC group, with less than 50% energy adequacy than in those with more than 50% energy adequacy (36.4% versus 9.5%, *p =* 0.009), as shown in Table 2.

Table 3 presents the results of logistic regression analysis for identifying the predictors of 90-day mortality in the total participants and the high mNUTRIC group. In the total participants, the univariate analysis revealed that age, mechanical ventilation, mNUTRIC scores, and energy adequacy of less than 50% were significantly associated with 90-day mortality. However, none of the above variables showed significant associations with 90-day mortality in the multivariate analysis. In the high mNUTRIC group, SOFA scores, mNUTRIC scores, and energy adequacy of less than 50% showed significant associations with 90-day mortality in univariate analysis. After multivariate analysis, the higher mNUTRIC scores (OR = 2.548, 95% CI: 1.177–5.514, *p =* 0.018) and energy adequacy of less than 50% (OR = 6.427, 95% CI: 1.674–24.674, *p =* 0.007) were revealed as significant predictors of 90-day mortality.

To assess the effectiveness of NST implementation for postoperative patients, we performed a comparative analysis of the nutritional status and clinical outcomes according to NST implementation (Table 4). The proportion of NST implementation was 29.8% (39 patients) in the low mNUTRIC group and 50.7% (38 patients) in the high mNUTRIC group. In the low mNUTRIC group, an average of 12.4 ± 6.7 kcal/kg/day of energy and an average of 0.55 ± 0.3 g/kg/day of protein was delivered to the patients. No differences in energy adequacy were observed between the groups who had or did not have NST implementation (49.6% versus 41.6%, *p =* 0.09). However, in the high mNUTRIC group, there was a significant difference in the average energy delivered between the patients with NST (14.7 ± 6 kcal/kg/day) and without NST implementation (10.7 ± 5.4 kcal/kg/day, *p =* 0.003). Additionally, the mean energy adequacy was significantly higher in patients with NST implementation than in those without NST implementation (58.8% versus 42.8%, *p =* 0.003).

## 4. Discussion

In this study, a large proportion (36.4%) of surgical ICU patients who underwent abdominal surgery exhibited a risk of malnutrition, as shown by high mNUTRIC scores (≥5). The high mNUTRIC group showed higher rates of postoperative complications, 90-day mortality, and more prolonged hospital stays. Additionally, increasing mNUTRIC scores and energy adequacy of less than 50% were independent predictors of 90-day mortality in the high mNUTRIC group.

Malnutrition is common in critically ill patients, with a prevalence of 39–50% [10,16,21]. The pathophysiology of malnutrition in surgical ICU patients is multifactorial. It is associated with catabolic hormones, cytokines responding to surgical stress, certain environmental factors such as restrictions in food intake, limitations in physical activity, and sedative drugs [16]. It can provoke the loss of muscle strength, impede wound healing, and increase the rate of infection, which are major contributors to the high risk of morbidity and mortality [1,11,16,22,23]. Moreover, surgical trauma could further increase the energy and protein requirements by creating a hypermetabolic and catabolic state that is commonly observed in post-surgical patients [23]. Thus, ASPEN and Society for Critical Care Medicine guidelines recommend that nutritional assessment should be performed on all postoperative patients in the ICU [4]. However, conventional screening tools such as anthropometric measurements of the body mass index or tissue fold thickness might not accurately reflect a patient’s nutritional status in the acute postoperative period [2,24]. During this phase, the bodyweight of critically ill patients is easily affected by tissue edema, massive fluid resuscitation, or the significant loss of lean body mass. In this study, we stratified the patients by an mNUTRIC score of 5 to identify surgical patients with a high risk of malnutrition. One-third of the total patients were classified in the high mNUTRIC group, and a significant difference was observed in 90-day mortality between the low and high mNUTRIC groups. These results are comparable with those of previous studies reporting a significant association between high mNUTRIC scores and 28-day mortality [2,13,21]. Interestingly, unlike most studies on medical patients with mechanical ventilation, our results suggest that the mNUTRIC score can also be useful in assessing nutritional risk in critically ill surgical patients after abdominal surgery without mechanical ventilation. As described above, it might be useful as a tool to simply measure the nutritional risk factors related to mortality in the acute postoperative period. In fact, we also analyzed about 30-day and 60-day mortality but failed to find a significant association with mNUTRIC score, and the only significant relationship was seen in 90-day mortality. Moreover, 90-day mortality is considered a more appropriate outcome measure for analyzing the effect of nutritional status on patient prognosis after abdominal surgery because it can minimize the bias effect of surgery-related complications in the acute phase after surgery, such as bleeding. Therefore, in this study, 90-day mortality was selected as the outcome measurement, and the 90-day mortality was finally specified in the manuscript.

Furthermore, setting the appropriate target for nutrient supplementation for patients at high risk of malnutrition is crucial in clinical practice. ASPEN and ESPEN guidelines suggest achieving more than 70% of the resting energy expenditure using indirect calorimetry. Nevertheless, most patients do not reach the targeted energy, and larger gaps between prescribed nutrition and actual delivery to the patients are commonly observed in the ICU setting. In a study by Assis et al. [25], only 63% of the prescribed energy was given to both medical and surgical patients, and a study by Chapple et al. [26] reported that only 39% of the prescribed energy was delivered to traumatic brain injury patients in the ICU. The most common reasons for low energy delivery were the interruption of feeding due to invasive interventions and bedside procedures [5,27]. Infusion of a lower than prescribed volume due to the patient’s intolerance, delays in initiating feeding in the ICU, or inaccuracy in feeding pump systems can also be significant factors associated with the gap between prescribed nutrition versus administered energy [25,28].

Even for surgical patients, achieving nutritional targets as suggested in the conventional guidelines can be difficult. Enteral nutrition might not be feasible in all cases, especially immediately after surgery due to bowel discontinuity, bowel ischemia, obstruction in the gastrointestinal tract, or ongoing peritonitis. These characteristic features in the acute postoperative period could result in a larger gap between the guidelines and clinical practice. Consequently, the nutritional therapy approach for surgical patients should be different from that of medical patients, and setting a new ideal target for nutrition support might be necessary. As the subject of this study, the patient who entered the surgical ICU after abdominal surgery will need to reach adequate energy consumption earlier than general medical critically ill patients due to the characteristics of the surgery (i.e., wound healing and a large amount of tissue and fluid loss that occurred rapidly during a short period of time during the surgery). Our results reveal that the cutoff value of energy adequacy for patients in the acute postoperative period was 50%. It was an independent prognostic factor associated with 90-day mortality in both univariate and multivariate analyses. We suppose that setting a lower target energy adequacy could be possible concerning permissive hypocaloric nutrition, as hypocaloric nutrition is preferred over isocaloric nutrition for the first week of ICU stay [3].

Regarding NST implementation, the incidence of NST implementation did not differ between the low and high mNUTRIC groups in our study. However, more energy and protein delivery was seen in the high mNUTRIC group with NST implementation. Additionally, the mean energy adequacy level in the group with NST implementation was higher than 50%, which was found to be a significant predictor of 90-day mortality, but not in the group without NST implementation. Nutritional support for postoperative patients who are undergoing complex physiological changes should be more fastidious. Our results suggest that NST implementation had an association with improved outcomes of post-surgical patients by effectively coming closer to the target nutritional goal and providing a high quality of nutritional care.

We obtained all 30-, 60-, and 90-day mortality values and analyzed the association with nutritional status based on the mNUTRIC score. The 90-day mortality was chosen based on a previous large study [29] and to minimize the confounding effect of surgery-direct related complications. As the most significant result was seen in 90-day mortality, only 90 days are specified in the text.

Despite these interesting findings, our results should be interpreted with caution due to their inevitable limitations. First, we simply calculated the nutritional requirement by an equation using bodyweight according to the ASPEN and ESPEN guidelines, not by indirect calorimetry, because most patients in our study were not on mechanical ventilation. Most surgical patients were not on mechanical ventilation. Second, our study included a small number of cases in a single institution. Additionally, we could not exclude the risk of selection bias in deciding whether to implement NST or not. It was mainly decided by the clinician’s judgment, not by a standardized protocol. Third, in terms of the number of covariates in the multivariable models, our data contain fewer than ten events for each variable entered into a logistic regression model. However, the validity of this rule of thumb has been questioned, and some studies showed this rule can be relaxed down to five events for each covariate [30,31]. Lastly, we did not include patients who had organ failure, such as hepatic or renal failure. Considering that the target energy and protein supply may vary depending upon kidney or liver function, it is necessary to analyze the target for subdivided nutritional supply according to the degree of organ impairment, including patients with organ failure, in the next study. If several confounders are managed well and the correct indicator group is selected, we could further increase the results of AUC and diagnostic abilities.

Further, we believe that a multicenter randomized controlled study with a large number of cases should be conducted in the future to confirm the results of this study. However, to the best of our knowledge, this was the first study to validate the optimal cutoff value of mNUTRIC scores and energy adequacy in critically ill patients after abdominal surgery in the ICU. We believe that our results provide evidence for setting appropriate nutritional targets and delivering an adequate amount of nutrition in the acute postoperative phase, which would ultimately improve the prognosis of surgical patients.

## 5. Conclusions

The majority of postoperative surgical patients were at risk of malnutrition according to mNUTRIC scores and received much less energy than expected. Identifying patients with mNTURIC scores of greater than 5 and supporting them with nutritional adequacy of more than 50% during an ICU stay would be useful in improving postoperative outcomes such as 90-day mortality.

## Figures and Tables

**Table 1:** Characteristics and clinical outcomes of total patients and according to the mNUTRIC score.

Variables	All Patients (*n* = 206)	Low mNUTRIC Group (mNUTRIC < 5)	High mNUTRIC Group (mNUTRIC ≥ 5)	*p*-Value
	*n =* 206	*n =* 131 (63.6%)	*n =* 75 (36.4%)	
Demographics				
Age (years)	62.5 ± 15.4	59.4 ± 15.3	67.8 ± 14	<0.001
Gender (male, %)	143 (69.4)	95 (72.5)	48 (64)	0.212
Body mass index (kg/m^−2^)	23.6 ± 4.5	23.8 ± 4.5	23.3 ± 4.5	0.483
Use of vasopressors (%)	64 (31.1)	25 (19.1)	39 (52)	<0.001
SOFA score	5.8 ± 3.7	4.3 ± 2.9	8.6 ± 3.5	<0.001
mNUTRIC score	4 ± 1.9	2.8 ± 1.1	6 ± 1	<0.001
Postoperative complication (%)	54 (26.2)	27 (20.6)	27 (36)	0.021
90-day mortality (%)	30 (14.6)	14 (10.7)	16 (21.3)	0.042
Length of ICU stay (days)	6.7 ± 6.2	5.5 ± 4.5	8.9 ± 7.9	0.001
Length of hospital stay (days)	32.3 ± 18.9	29.2 ± 17	37.6 ± 20.9	0.003
Type of nutrition patients received (%)				0.929
PN	40 (19.4)	27 (20.6)	13 (17.3)	0.715
EN	9 (4.4)	6 (4.6)	3 (4)	1.000
PN + EN	20 (9.7)	12 (9.2)	8 (10.7)	0.808
Oral diet	137 (66.5)	86 (65.6)	51 (24.8)	0.761
Implementation of NST	77 (37.4)	39 (29.8)	38 (50.7)	0.004
Laboratory test				
Total protein (g/dL)	5 ± 0.8	5.4 ± 1.1	4.8 ± 0.9	<0.001
Albumin (g/dL)	3 ± 0.4	3.2 ± 0.7	2.9 ± 0.7	0.001
Prealbumin (mg/dL)	16.2 ± 7.4	15.6 ± 7.8	11 ± 6.7	0.009
Transferrin (mg/dL)	130.4 ± 38.4	147.1 ± 55.5	114.2 ± 38.9	<0.001
Total cholesterol (mg/dL)	106.6 ± 41	108.6 ± 42.9	79.1 ± 30	<0.001
HDL cholesterol (mg/dL)	23.7 ± 10.9	28.3 ± 12.8	19.6 ± 9.5	<0.001
LDL cholesterol (mg/dL)	57.7 ± 25.9	57.3 ± 26.6	40 ± 19.6	<0.001

SOFA = sequential organ failure assessment, mNUTRIC = modified Nutrition Risk in Critically Ill, ICU = intensive care unit, PN = parenteral nutrition, EN = enteral nutrition, NST = nutrition support team, HDL = high-density lipoprotein, LDL = low-density lipoprotein.

**Table 2:** Nutritional and clinical outcomes according to different nutritional adequacy cutoff values in the high mNUTRIC group (mNUTRIC ≥ 5). (A) Energy adequacy < 50%, (B) energy adequacy < 60%, and (C) energy adequacy < 70%.

Variables	All Patients	Energy Adequacy < 50%	Energy Adequacy ≥ 50%	*p*-Value
	*n =* 75 (100%)	*n =* 33 (44%)	*n =* 42 (56%)	
(A) Energy adequacy < 50%				
Postoperative complication (%)	27 (36)	13 (39.4)	14 (33.3)	0.634
90-day mortality (%)	16 (21.3)	12 (36.4)	4 (9.5)	0.009
Length of ICU stay (days)	8.9 ± 7.9	9.2 ± 7.7	8.6 ± 8.2	0.763
Length of hospital stay (days)	37.6 ± 20.9	36.8 ± 17.2	38.3 ± 23.5	0.756
(B) Energy adequacy < 60%				
Postoperative complication (%)	27 (36)	19 (36.5)	8 (34.8)	1.000
90-day mortality (%)	16 (21.3)	12 (23.1)	4 (17.4)	0.762
Length of ICU stay (days)	8.9 ± 7.9	8.6 ± 7.4	9.5 ± 9.1	0.660
Length of hospital stay (days)	37.6 ± 20.9	36.4 ± 19.9	40.4 ± 23.2	0.459
(C) Energy adequacy < 70%				
Postoperative complication (%)	27 (36)	22 (34.4)	5 (45.5)	0.511
90-day mortality (%)	16 (21.3)	14 (21.9)	2 (18.2)	1.000
Length of ICU stay (days)	8.9 ± 7.9	8.7 ± 7.4	9.9 ± 10.8	0.640
Length of hospital stay (days)	37.6 ± 20.9	36.6 ± 2.5	44 ± 24.5	0.277

ICU = intensive care unit.

**Table 3:** Univariate and multivariate analysis of risk factors for 90-day mortality (A) in the total participants and (B) in the high mNUTIRC group (mNUTRIC score ≥ 5).

Variable	Univariate		Multivariate	
	OR (95% CI)	*p*	OR (95% CI)	*p*
(A) Total participants				
Age	1.034 (1.006–1.064)	0.017	1.021 (0.990–1.053)	0.180
Use of vasopressor	1.590 (0.715–3.534)	0.255		
Mechanical ventilation	3.757 (1.684–8.380)	0.001	2.327 (0.939–5.765)	0.068
SOFA score	1.099 (0.991–1.219)	0.074		
mNUTRIC score	1.499 (1.198–1.875)	<0.001	1.215 (0.793–1.859)	0.371
Energy adequacy < 50%	4.333 (1.928–9.737)	<0.001	1.389 (0.299–6.449)	0.675
(B) High mNUTRIC group (mNUTRIC score ≥ 5)				
Age	1.022 (0.980–1.066)	0.308		
Use of vasopressor	0.903 (0.299–2.728)	0.857		
Mechanical ventilation	2.431 (0.779–7.582)	0.126		
SOFA score	1.067 (0.903–1.261)	0.049	0.935 (0.752–1.164)	0.547
mNUTRIC score	2.108 (1.143–3.885)	0.017	2.548 (1.177–5.514)	0.018
Energy adequacy < 50%	5.429 (1.554–18.963)	0.008	6.427 (1.674–24.674)	0.007

SOFA = sequential organ failure assessment, mNUTRIC = modified Nutrition Risk in Critically Ill.

**Table 4:** Average calorie and protein delivered according to NST implementation (A) in the low mNUTRIC group (mNUTRIC score < 5) and (B) the high mNUTRIC group (mNUTRIC score ≥ 5).

Variables	All Patients	NST Implementation (+)	NST Implementation (−)	*p*-Value
	*n =* 131 (100%)	*n =* 39 (29.8%)	*n =* 92 (70.2%)	
(A) Low mNUTRIC group (mNUTRIC score < 5)				
Average energy delivered (kcal/kg/day)	11 ± 5.1	12.4 ± 6.7	10.4 ± 4.2	0.09
Average protein delivered (g/kg/day)	0.51 ± 0.24	0.55 ± 0.3	0.49 ± 0.2	0.224
Energy adequacy (%)	44 ± 20.5	49.6 ± 26.9	41.6 ± 16.7	0.09
(B) High mNUTRIC group (mNUTRIC score ≥ 5)				
Average energy delivered (kcal/kg/day)	12.7 ± 6	14.7 ± 6	10.7 ± 5.4	0.003
Average protein delivered (g/kg/day)	0.58 ± 0.28	0.66 ± 0.26	0.49 ± 0.27	0.009
Energy adequacy (%)	50.9 ± 24	58.8 ± 23.9	42.8 ± 21.6	0.003

NST = nutrition support team.

## Data Availability

The datasets used and/or analyzed during the current study are available from the corresponding author on reasonable request.
